# A Review on Properties of Natural and Synthetic Based Electrospun Fibrous Materials for Bone Tissue Engineering

**DOI:** 10.3390/membranes8030062

**Published:** 2018-08-14

**Authors:** Deval Prasad Bhattarai, Ludwig Erik Aguilar, Chan Hee Park, Cheol Sang Kim

**Affiliations:** 1 Department of Bionanosystem Engineering, Graduate School, Chonbuk National University, Jeonju 561-756, Korea; devalprasadbhattarai@gmail.com; 2 Department of Chemistry, Amrit Campus, Tribhuvan University, Kathmandu 44613, Nepal; 3 Division of Mechanical Design Engineering, Chonbuk National University, Jeonju 561-756, Korea

**Keywords:** bone tissue regeneration, electrospinning, biocompatible polymers, nanotechnology, biomimicry

## Abstract

Bone tissue engineering is an interdisciplinary field where the principles of engineering are applied on bone-related biochemical reactions. Scaffolds, cells, growth factors, and their interrelation in microenvironment are the major concerns in bone tissue engineering. Among many alternatives, electrospinning is a promising and versatile technique that is used to fabricate polymer fibrous scaffolds for bone tissue engineering applications. Copolymerization and polymer blending is a promising strategic way in purpose of getting synergistic and additive effect achieved from either polymer. In this review, we summarize the basic chemistry of bone, principle of electrospinning, and polymers that are used in bone tissue engineering. Particular attention will be given on biomechanical properties and biological activities of these electrospun fibers. This review will cover the fundamental basis of cell adhesion, differentiation, and proliferation of the electrospun fibers in bone tissue scaffolds. In the last section, we offer the current development and future perspectives on the use of electrospun mats in bone tissue engineering.

## 1. Introduction

Tissue engineering is an interdisciplinary field in which engineering principles are applied for the development of biologic functional substitutes in body that restores, maintains, or improves tissue functions [1,2]. It uses basic chemistries to manipulate cell fate in a supporting matrix, called scaffold, which offers mechanical as well as biological influences to support migration, attachment, proliferation, and integration of cells to form tissues [3]. A scaffold is a three-dimensional substrate acting as template in tissue engineering [4]. Some prerequisite criteria for a successful design of suitable scaffolds are three-dimensional structure and conducive mechanical properties for physical support, high surface area for cellular attachment, biomimetic framework for guiding new tissue formation, and biocompatibility for complying host responses towards the construct scaffolds [5,6]. Various designs of scaffolds have been developed to mimic the composition and microstructure of extracellular matrix (ECM), with hierarchical structures ranging from nanometer to millimeter scales [4,7]. Bone tissue scaffolds may be metallic [8], hydrogels [9,10,11], or electrospun nano/microfibers [12,13,14]. Commonly used metallic bio-implants includes stainless steel [15,16], titanium [17] or its alloys, nitinol, etc. [18].

From structural point of view, natural extracellular matrix (ECM) having various interwoven protein fibers with diameters ranging from tens to hundreds of nanometers offers a background to foster cell growth. The ECM limits the tissue boundary, biomechanical properties, and cell polarity. The composition of ECM varies with the nature of tissues. However, the common composition of ECM involves structural proteins (e.g., collagen, elastin), adhesive proteins (e.g., fibronectin, laminin), and proteoglycans-protein-polysaccharides complex. The common and major constituents of bone tissue are collagen and hydroxyapatite [19].

Among the various processing techniques, like phase separation [20,21], self-assembly [22,23,24,25], fused deposition modeling [26], and electrospinning [27,28,29,30,31], etc. for the fabrication of nanofibrous scaffolds to be used as ECM substitutes, the electrospinning process is the most promising and versatile technique mainly due to its mimicking properties to the physical dimensions of natural fibrillar ECM [32], processing versatility to a wide range of materials, controllable mechanical properties, and simple operation at low cost. The large surface area of electrospun fibers and their porous morphology favors the cell adhesion, proliferation and differentiation. If required, such electrospun fibers can further be surface functionalized by incorporating biomolecules, like DNAs [33], growth factors [34], etc., to better control the seeded cell proliferation, differentiation, and integration on the scaffold. All of these properties may lead to improvements to offer a more robust biomimetic microenvironment to the developing tissues. The electrospun nanofibers have also attracted tremendous attention in the fabrication of bone tissue scaffolds identifying suitable material compositions and using them in electrospinning. The nanofiber substratum may provide a good platform for bone cells adherence and growth in a similar manner to those in other tissues. Further osteoblastic differentiation and mineralization have also been reported to be regulated satisfactorily on the nanofibrous surface [35]. The electrospun nano/micro fibers find its use in tissue engineering in dentistry. Chieruzzi, M., et al. have mentioned the role of natural and synthetic polymers or composites or hybrid materials, natural or synthetic ceramics useable as self-setting synthetic bone graft materials. In addition to cartilage and bone tissue, biomaterials that are used in the formulation of soft scaffolds or hydrogel are expected to guide the formulation of tissues of pulpodetinal complex and periodontal complex [36].

This article gives a brief overview of current works on development of scaffolds for bone tissue engineering based on electrospinning technique. First, we present a brief introduction of bone morphology and basic principle in bone tissue engineering. We then describe the electrospinning technique relating their fabrication, advantages, and applications in bone tissue engineering. We will also mention about the polymers that are used in electrospinning for bone scaffolds preparation in current years. Finally, we give conclusion along with perspectives and challenges of biomimetic scaffolds for bone tissue engineering based on electrospun nanofibers.

## 2. Native Bone Structure and Composition

### 2.1. Composition of Bone

Ideally, synthetic bone tissue scaffolds are expected to mimic both the structures and functions of natural bone besides having good mechanical properties and biocompatibility. Therefore, it becomes necessary and relevant to take know-how about physicochemical architecture of native bone along with pertinent biomechanical and biochemical properties to gain an insight into choosing and harnessing the best biomaterial types that are used in the fabrication of bone tissue scaffolds. Bone is regarded as a complex, highly organized, and specialized connective tissue that is composed of nearly 70% inorganic constituents (primarily, hydroxyapatite (HA), Ca_10_(PO_4_)_6_(OH)_2_) [37], nearly 20% organic constituents and 10% water. Among many organic constituents, the type I collagens is most abundant (nearly 90%), which anchors HA crystals and also enhances cellular attachment due to its abundant RGD (Arg-Gly-Asp tripeptide sequence) residues [38]. Bone plays a pivotal role in specific functions in human physiology, such as supporting body and vital organs, movement, blood production, minerals storage, homeostasis, housing for multiple progenitor cells, such as mesenchymal, hemopoietic stem cells, and so on.

### 2.2. Cellular Organization and Bone Remodeling

Bone is a dynamic tissue that undergoes constant remodeling throughout the life resulting into a balance between the worn out of old mineralized bone and formation of new bones [39]. Bone remodeling or repairing process involves osteogenic cells that have very specific roles and functions. These cells are osteoblasts, osteocytes, osteoclasts, and bone lining cells.

#### 2.2.1. Osteoblast (Bone Forming Cells)

Osteoblasts are bone building cells. They secret collagen matrix and are involved in protein synthesis, such as osteopontin, osteocalcin, etc. The precursors of osteoblasts are multipotent mesenchymal stem cells (MSCs) [40,41]. Osteoblasts secrete organic and inorganic components of bone ECM, including type I collagen, proteoglycans, glycoproteins and γ-carboxylated proteins, bone sialoprotein, osteopontin, osteocalcin, osteonectin, cytokines, insulin like growth factor I, II, transforming growth factor (TGF-β), bone morphogenic proteins (BMPs), and alkaline phosphatase [42,43]. After active bone formation, osteoblasts are thought to have either of three potential fates: the first is, the osteoblasts may become a quiescent cell (lining cells) on the surface of bone, the second is the osteoblasts may undergo programmed cell death (apoptosis), and the third one is the cells that may embed in its own osteoid and differentiate into osteocytes. Cells of the osteoblast lineage provides the factors essential for the differentiation of osteoclasts (bone-resorbing cells). The newly formed bone matrix is yet to be calcified, which is called as osteoid. 

#### 2.2.2. Osteoclasts (Bone Resorption Cells)

Osteoclasts are involved in the absorption and removal of old bone cells and can be regarded as bone removing cells. The precursors of osteoclasts are hematopoietics cells of monocyte or macrophage lineage [41]. Osteoclasts are multinucleated giant cells with ruffled border region of the cell membrane that are surrounded by organelle-free region. They anchor to the bone surface with the help of integrins. Hematopoietic cells of the monocyte macrophage differentiate into osteoclast precursors in the vicinity of osteoblast-lineage cells. The osteoblast lineage cells mediate the action of bone resorption stimulating factors to induce osteoclasts differentiation from osteoclast precursors [44].

#### 2.2.3. Osteocytes

Osteocytes are terminally differentiated osteoblasts cells that are capable of sending signal based on stress and strain felt therein. These are the most abundant cells type in bone [41]. Osteocytes reside in lacunae within the mineralized bone matrix and send their dendritic projection through tiny tunnels (canaliculi) to connect to the bone surface and vasculature. Osteocytes are crucial cells for the normal functioning and maintaining homeostasis in the adult skeleton. These cells play multifunctional roles in coordinating bone remodeling by regulating both osteoblast and osteoclast function [45]. Osteocytes may function in an endocrine manner to regulate phosphate homeostasis.

#### 2.2.4. Bone Lining Cells

Bone lining cells are the cells of bone surface that direct mineral uptake and release in bone [46]. Bone tissue is maintained by the balance of two opposing processes; bone formation and bone resorption. Osteoblast-lineage cells (osteoblasts, osteocytes, and bone lining cells) are involved in bone formation, while osteoclasts are involved in bone resorption. However, researches have shown that osteoblast-lineage cells are not only involved in bone formation, but are also involved in bone resorption supporting the differentiation and activation of osteoclasts [47]. Imbalance between bone formation and bone resorption may cause some bone related deformities, like osteoporosis [48].

### 2.3. Hierarchical Bone Structure

**Figure 1:**
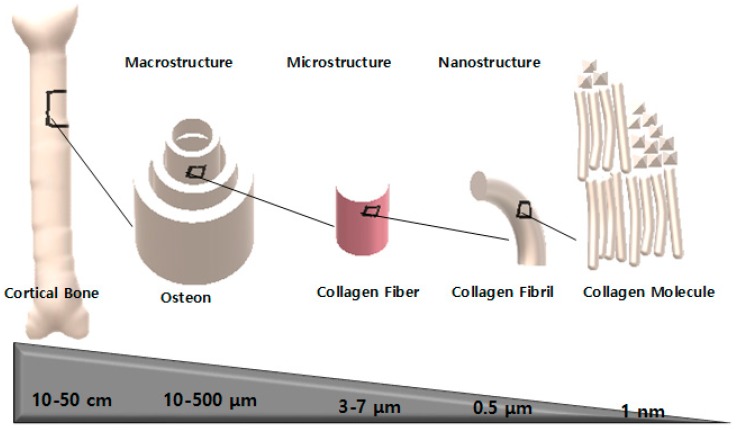
Schematic of the hierarchical macrostructure constructs of cortical bone, the regular and cylindrical shaped of osteon constructs, and microstructure/nanostructure of collagen fibers/fibrils/molecule.

Bone is a complex living tissue in which different geometrical features occur on various length scales ranging from nanometer scale to the whole structure of bone imparting a hierarchical architecture design. The hierarchical structure of bone can be studied in terms of macro-, micro-, and nano- or some intermediate structures falling within these scales (Figure 1).

#### 2.3.1. Macrostructure

The compact (cortical) bone and cancellous (trabecular/spongy) bone are macrostructure in bone tissue, which differ by the degree of porosity or density. The microstructure of these bones determines the fate of histological difference. Irregular and sinuous convolutions of lamellae construct microstructure of cancellous bone while regular and cylindrical shaped of lamellae constructs microstructure of cortical bone, resulting in the relatively lower density of cancellous bones [49].

#### 2.3.2. Microstructure

Mineralized collagen fibers form into planar sheets called lamellae (3–7 µm wide). In some cases, 3–8 lamellae wrap in concentric layers around central canal to form osteon or Haversian system, which are cylindrical tubes with about 200–250 µm in diameter running roughly parallel to the long axis of bone.

#### 2.3.3. Nanostructure

The prominent structure that is seen at the nanoscale of bone tissue is the collagen fibers surrounded and infiltered by minerals. The crystals and collagen fibrils are down to ten nanometers, preferably called sub-nanostructure, which consist of crystals, collagens, and non-collagenous organic proteins. The crystals are of calcium phosphate (the most common are hydroxyapatite and β-Tricalcium Phosphate, β-TCP) and the most common collagen is type I collagen. The non-collagenous organic proteins are osteopontin, sialoprotein, osteonectin, and osteocalcin. These non-collagenous proteins regulate the size, orientation and crystal habit of the deposited minerals. These proteins may serve as a reservoir for calcium and phosphorous ions by chelation. The mass ratio of calcium to phosphorous in hydroxyapatite crystal is 1.6. The hierarchical scales of bone tissue are mentioned in Table 1 and the physical properties of bone components is mentioned in Table 2.

### 2.4. Biomimicking and Bone Tissue Engineering

Tissue engineering is an application of engineering principle for the preparation of bone tissue scaffolds to restore, maintain, and improve tissue functions. Bone injury and deformities arising from trauma, malformation, osteogenesis imperfecta, osteoarthritis, osteomyelitis osteoporosis, or due to orthopedic surgery, such as total joint arthroplasty, spine arthrodesis, implant fixation, or due to primary tumor resection and tumors present a difficult challenge in effective treatment. Mal-union or non-union problems in bone tissue create a serious problem in natural healing process, especially in the case of serious fractures, defect or in elderly patients. The non-union or bone is unable to heal spontaneously within a patient’s life time (critical size bone defect) necessitates substitutionary materials from different site bone of the same person (autograft) [53] or from other humans or from cadavers (allograft) [54] or from non-human (xenograft) [55] mechanism to fill the bone defect. Commonly adopted path in solving such problems is an invasive surgery to align and stabilize the bone with metallic pins, screws, plates, or rods. Use of metallic implants offers some appealing benefits, such as providing mechanical strength and integrity. However, it is not free from flaws to some inherent problems like stress shielding, stiffness, infections and chronic pains. These facts necessitate an improved clinical therapy in relation to the bone tissue engineering, such that an engineered implant would reduce the need for multiple surgeries that are related to the removal of metallic stabilizers and graft harvesting. Among many alternatives for the fabrication of bone tissue scaffolds, electrospinning is a promising and versatile technique to develop nanofibrous scaffold that would meet the basic and fundamental requirements, such as high porosity, mimicking the native bone extracellular matrix for potential applications as bone tissue engineering scaffolds to restore the degenerated function of bone while using natural or synthetic or blends of both types of polymers such that the scaffold should be structurally sound to withstand the mechanical stresses during tissue neo-genesis [56,57].

**Figure 2:**
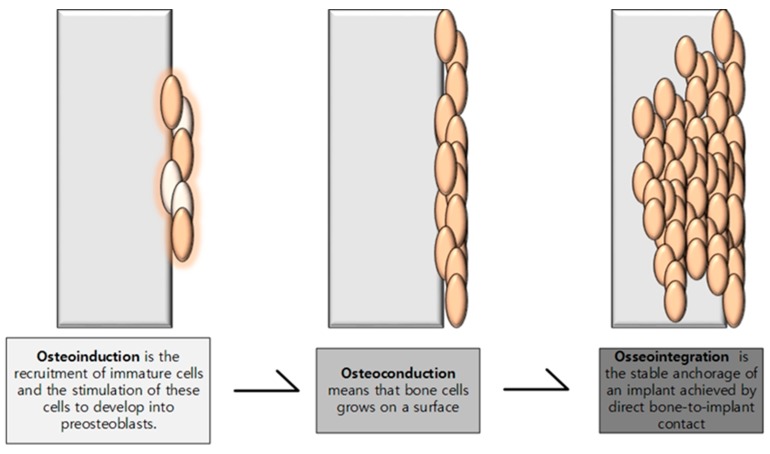
Schematic representation of osteoinduction, osteoconduction, and osseointegration process in bone tissue regeneration [62].

The basic concept behind tissue engineering is the setting of tissue scaffolds in the niche of biological responses of local environment within body in conjunction with engineering principles [58]. The primary purpose of scaffolds that are used in tissue engineering is to incite and promote the natural healing process of defected bone [59]. For this purpose, the synthesized scaffolds should mimic the physicochemical environment of body and would degrade as native tissues integrate and actively promote the desirable physiological responses or to prevent undesirable physiological responses. The scaffold is expected to provide temporary mechanical support to the affected area. The porous architecture of scaffold should meet the criteria for vascularization and bone in-growth [60]. The scaffold should boost up the adherence and proliferation of bone cells along with the formation of extracellular matrix on the surface or pores of scaffold (osteoconduction) [52]. Furthermore, the scaffold should trigger the formation of new bones or osteogenic differentiation via biomolecular signaling and promoting osteodifferentiation (osteoinduction) [61,62]. The scaffolds should enhance cellular activity towards scaffolds-host tissue integration that integrates with native tissue and fills the void or defect (osteointegration) [63,64]. The schematic representation of osteoinduction, osteoconduction and osteointegration is shown in Figure 2. In some cases, the scaffolds may be loaded with drugs [65] or bioactive molecules [66] to deliver such loaded moieties in controlled way to accelerate the healing process. In practice, bone tissue engineering implements single or combination of different strategies among cell transplantation, acellular scaffolds, gene therapy, stem cell therapy, and growth factor delivery.

The challenges in tissue engineering are to design and fabricate temporary bone scaffolds that could deliver the bioactive molecules and drugs to the injured sites to enhance the biological functionality. The scaffold should be biocompatible and should promote cell function, such as attachment, proliferation, migration, and differentiation. Furthermore, the degraded products of scaffold should be non-toxic, non-inflammatory, and could be easily eliminated from the body. The large volume fraction of scaffold facilitates the cell migration and transport of nutrients, as well as regulatory factors, such as growth factors, hormones, etc. [67].

#### Biomimetic

Mimicking the geometric architecture of natural bone in a synthetic scaffold can promote favorable cellular activities through the controlled delivery of biospecific cues. Selection of polymers for bone scaffolds should meet some criteria, such as: the polymer degradation rate is to be concomitant to tissue in growth rate, the biodegraded products should be biocompatible and polymer processability should be simple [68]. Therefore, some key criteria, such as processability, mechanical property, morphology, porosity, hydrophilicity, degradability, and biocompatibility must be fulfilled while selecting a polymer for bone tissue scaffolds preparation. Bone tissue engineering aims at growing ECM via seeding bone cells on synthetic scaffolds. Bone tissue scaffolds can be made by various techniques, such as electrospinning [69], hydrogel gel formation method [70,71], electrochemical anodization of metal [17], and thermally-induced phase separation method [72]. The commonly used biomaterials in bone tissue scaffolds are β-TCP ceramics [73], hydroxyapatite [74,75], bioactive glass [76], metal or alloy [77], and nano/micro fiber-based scaffolds. Bone tissue scaffolds that are prepared from fibrous materials are highly desirable. Mostly nanofibers are prepared by an electrospinning technique that enables the fabrication of scaffolds with fiber diameter ranging from nanometers to micrometers mimicking the physico-chemical properties to that of natural ECM. High porosity, pore interconnectivity, large surface area, surface topography, and hydrophilicity provide suitable surface sites for bone cells to adhere, proliferate and grow on the scaffold. Bone tissue has hierarchical organization over the length scales ranging from nano to micro structure of ECM. The following points should be noted while designing the biomimetic scaffold for bone tissue engineering; (i) the scaffold should mimic the nanofibrous collagen ECM; (ii) the scaffold should be porous sufficient to allow for the cell ingrowth and efficient transport of oxygen, nutrition, growth factors, as well as cellular waste products; and, (iii) mechanical strength of scaffold should be sufficient to withstand the mechanical stress during tissue neogenesis. The pore size of scaffold should be within critical range of lower bound determined by cell size and upper bound determined by binding sites or specific surface area. For bone tissues, the pore size of scaffolds is expected to fall between 100–500 µm [78,79]. Also, the pore geometry should be conducive to cell morphology. Hierarchically, the overall scaffold should have sufficient mechanical integrity to handle in surgery and the rate of scaffold degradation should be comparable to the rate of tissue formation.

### 2.5. Polymers Used in Bone Tissue Engineering

Polymers are used in controlled delivery of bioactive molecules in bone tissue scaffold due to their hydrolytically unstable linkage or enzymatically degradable linkage in the backbone and tunable biodegradation rate [80]. Ceramic materials have also good biodegradability and good bioactive molecules releasing potency at a controlled rate, but lack enough mechanical strength. Natural polymers, such as collagen, alginate, fibrin, and gelatins are useful in bone tissue engineering for drug delivery purpose, but lack load bearing enough mechanical strength and appropriate degradation rates. To overcome such problems, bioactive molecules can be either covalently bound or entrapped inside such polymer matrixes or ceramics can be coated with polymer for drug delivery purpose [81]. Degradation of polymers during the course of physiological action results into the release of bioactive molecules or drugs. Polymers that undergo degradation under physiological conditions are termed as biodegradable polymers. The degradation chemistry of polymers depends upon the types and nature of polymers blends (pristine/copolymer), architectural scale (micro scale/nano scale), presence of hydrolytic accelerators or suppressors, etc. [82]. In vivo degradation rate of polyester is higher than the in vitro degradation rate more presumably due to optimum concentration of ester cleaving enzymes, such as lipase in human body [83].

## 3. Principle of Electrospinning

The electrospinning technique was first developed in the early 1900s [72]. Technologically, electrospinning is a physical process in which viscoelastic solution is squeezed into jet under high electrostatic forces overcoming the cohesive forces resulting into nano/micro sized fiber after the successive evaporation of solvent. In electrospinning setup, the nozzle serves as an electrode in which a high electric field is applied and the collector is made contact with counter electrode. The applied voltage deforms the drop of polymer solution into cone shape (Taylor cone) at the tip of nozzle, followed by flowing towards the direction of counter electrode. Due to electrostatic repulsion, the jets elongate continuously directing towards the collector under whipping motion. On the way, the solvent evaporates and dry solid fibers with diameters ranging from micrometer to nanometer are spun on the collector [84].

### 3.1. Factors Affecting the Electrospinning Process

Various parameters, such as polymer properties, solvent properties, solution properties, processing, and ambient conditions determine the fate of the electrospun fiber morphology [32]. Researches have shown that electrospun fiber diameter is directly related to concentration, molecular mass, viscosity, and flow rate of polymer solution. Increasing the electrical conductivity decreases the electrospun fiber diameter. The basic setup of electrospinning consists of a syringe pump, metallic needle, a ground collector and high voltage power supply. Polymer solution serves as fundamental electrospinning material and it is put into a syringe that is fitted on syringe pump. Cengiz, F., et al. investigated the effect of tetraethylammonium bromide salt on roller electrospinning of polyurethane nanofibers. They found increased conductivity, viscosity, and fiber diameter with increasing the salt concentration [85]. Geng, X., et al. investigated the effect of molecular weight of chitosan on its electrospinning fibers [86]. Demir, M.M., et al. studied the effect of electric field, temperature, conductivity, and viscosity of solution on the electrospinning process on polyurethaneurea co-polymer. They found increased fiber diameter with increased concentration of spinning solution. Low concentration of solution developed beads, increased concentration of fibers developed curly fibers, and the highest concentration of solution developed a trimodal distribution of fibers in diameter in their findings [87]. Thompson, C.J., et al. studied the effect of initial polymer concentration, density of solution, solvent vapor pressure, volumetric charge density, nozzle to collector distance, relaxation time and viscosity on the jet radius, and the consequent electrospun fibers [88]. Arayanarakul, K., et al. investigated that addition of sodium chloride and sodiumdodecylsulphate (SDS) on polyethylene oxide (PEO) solution increased conductivity with decreased surface tension, resulting in the total suppression of beads on electrospun mat [89]. Table 3 shows the effect of different parameters on the electrospun fiber morphology.

#### 3.1.1. Solvent Effect

Solvent used in electrospinning affect solution spinnability. Solvent properties, such as dielectric constant and boiling temperature can influence the electrospinning and material morphology. The effect of solvent on cellular responses has also been reported [98,99].

#### 3.1.2. Substrates Effect

The shape and morphology of electrospun fiber depends upon the polymer properties, such as glass transition temperature, solubility, composition of substrates used, and molecular weight/number average molar mass [100].

#### 3.1.3. Polymer Solution Properties

The electrospinning process is also affected by polymer solution properties, such as viscoelasticity, concentration, surface tension, and electrical conductivity [101].

#### 3.1.4. Ambient Factor

Relative humidity, vapor pressure of solvent, and temperature affect electrospinning [102]. 

#### 3.1.5. Operation Factor

The applied electric field, geometry of electrode, flow rate of solution, size of the nozzle tip, substrates used for collector, and the nozzle to collector distance affect the morphology of electrospun fiber [94,103].

Different types of electrospun fibers, such as aligned fibers and random fibers, can be produced by applying different techniques of electrospinning.

### 3.2. Electrospun Materials for Bone Tissue Engineering

Electrospinning produces fibers ranged from micrometer to few nanometers. Nano/micro fibrous materials have attracted considerable interest in tissue engineering field, including bone reconstruction mainly due to structural similarity to tissue ECM and processing accessibility to a wide range of materials. For best fitting, tissue engineering scaffolds should mimic the native extracellular matrix (ECM) in terms of both physical structure as well as chemical composition [104]. Varieties of nano/micro range spinning fibers have been developed to mimic the native extracellular matrix for potential application in bone tissue scaffolds (cell supporting matrixes) to restore the loss or diminished functionalities. Biodegradable polymers, bioactive inorganic materials, and their nanocomposites with suitable properties and conducive environment for osteoblasts and progenitor/stem cells have been developed. The surface functionalization of nanofibers with hydroxyapatite minerals and proteins/peptides chains, drug encapsulation within the nanofibers are some promising strategies to embrace the therapeutic functions with nanofibrous materials. Nanofiber materials impart good space for cells anchorage and successive spreading over the material in addition to triggering them for secreting appropriate ECM molecules that are targeted to corresponding tissue cells [43]. Such nanofibers regulate further osteoblastic differentiation and mineralization under the ambient condition.

### 3.3. Polymers Used in Electrospinning

**Figure 3:**
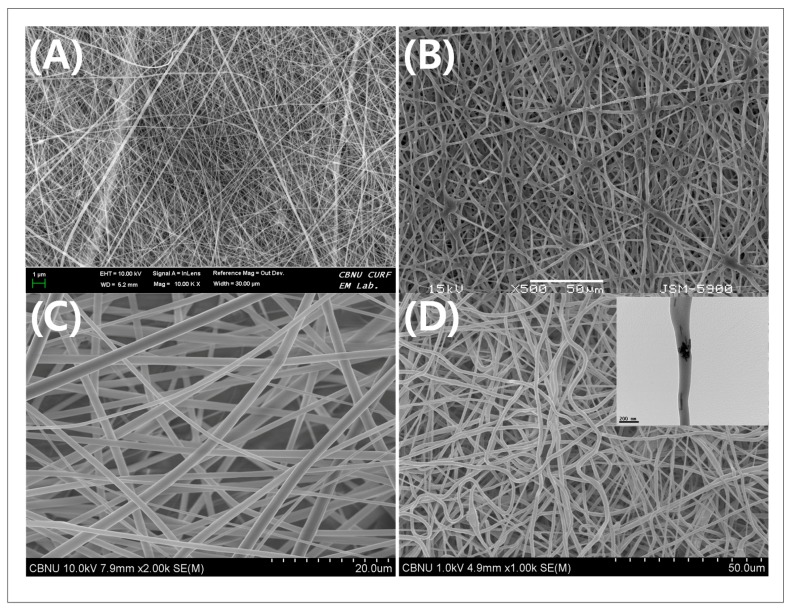
Electrospun nanofibers primarily used in bone tissue engineering. Field emission scanning electron microscope (FE-SEM) images examples of natural material (**A**) Silk Nanofiber; synthetic material (**B**) Polycaprolactone (PCL); *co*-polymer blends (**C**) PCL-Cellulose acetate; and, polymer-ceramic composite (**D**) PCL-Cellulose Acetate-β-TCP (inset: TEM image of calcium phosphate particles (β-TCP) inside the nanofiber) [57].

Polymers and polymer-ceramic composites materials have long been used for the fabrication of synthetic bone scaffolds due to their biocompatibility, tunable degradability, processability, and versatility. Cellular or enzymatic degradation pathways or exposure of physiological aqueous environment degrade the polymer scaffolds via hydrolysis [105,106]. The rate of polymer degradation can be tuned by copolymerization or blending the solution for electrospinning or changing the degree of hydrophobicity and crystallinity. The polymers that are used in electrospinning can be plainly classified into natural polymer, synthetic polymer, and polymer blends. Synthetic polymers exhibit better mechanical properties than natural polymer. Blending of two synthetic polymers or two natural polymers or preferentially synthetic polymer and natural polymer could be useful to develop an electrospun mat with enhanced desirable properties, such as mechanical strength or biocompatibility [107]. The field emission scanning electroscope (FE-SEM) image of some natural and synthetic fibers are shown in Figure 3.

### 3.4. Electrospun Scaffolds from Natural Polymer

The common natural polymers being used for bone tissue engineering are collagen, alginate, chitosan, silk, gelatin, elastin, glycosaminoglycan (GAG), etc. [108,109,110]. The natural polymers exhibit good biocompatibility but low mechanical properties when compared to synthetic polymers. Herein, we discuss some natural polymers in brief.

#### 3.4.1. Silk

Silk is a natural protein polymer composed of 70–80% fibroin (core protein) and 20–30% sericin (adhesive proteins) as major constituents [111,112]. It is obtained from cocoons of the larvae of silkworm (most commonly, from *Bombyx mori*) [113] or other insects [114]. Silk has been used as a biomedical material for suture in surgeries due to its remarkable mechanical properties, biocompatibility, tunable degradation rates in in vivo as well as in vitro. Silk fibroin has been used for several in vivo studies for bone, brain, subcutaneous, etc. [115]. Kirker-Head C., et al. have shown silk fibroin as osteoconductive matrix for healing critical sized mid femoral segment defects in nude rats [116]. HJ Jin, et al. used electrospun silk fibroin mats (average fiber diameter 700 ± 50 nm) for the study of human bone marrow stromal cell responses [117]. Wray, L.S., et al. studied the effect of degumming on silk fibroin protein in the issue of biocompatibility and reproducibility related to the material formation [112]. The toughness and mechanical strength, along with good biocompatibility of silk fibroin, makes it a material of choice in bone tissue engineering. Growth factors, like BMP-2, could be loaded in electrospinning solution to induce osteogenic differentiation of human bone marrow stromal cells when cultured [118]. Incorporation of hydroxyapatite nanoparticles into silk matrix has been shown to enhance the bone regeneration in a rabbit model [119]. Gao, Y., et al. found increased nucleation and growth of hydroxyapatite by the addition of tussah silk fibroin in poly(l-lactic-*co*-glycolic acid) in electrospinning. They also observed increased cytocompatibility, osteoblast differentiation, and mechanical properties of the composite scaffolds [120].

#### 3.4.2. Collagen

Collagen is the most abundant protein in the human body which forms a major constituent of ECM in various connective tissues. It has surface binding sites for cells and it is regarded as an excellent substrate for cell attachment. Its low young modulus value (0.8 GPa) can be increased by crosslinking with synthetic polymers. It is biocompatible and bioactive natural polymer that consists of 25% to 35% of the total body content. Chemically collagen protein is composed of a triple helix of polypeptide subunits, two identical chains (α1), and an additional chain having slightly different chemical composition (α2) [84]. Collagen containing nanofiber scaffolds have been fabricated by electrospinning using 1,1,1,3,3,3 hexafluoroisopropanol (HFIP) [121]. To avoid the handling of toxic HFIP solvent, a more benign alternative solvent mixture of formic acid and acetic acid can be used for PCL/gelatin or PCL/collagen. But, the change of solvent system is found to be associated with a bit different mat morphology and cellular response [98]. Dippold, D., et al. have fabricated polycaprolactone-collagen composite nanofibers by electrospinning using diluted acetic acid as solvents.

#### 3.4.3. Gelatin

Gelatin is a commonly used natural polymer that is derived from collagen. It is biocompatible and biodegradable. The surface charge of gelatin depends upon its processing method (acidic-processing or alkaline pre-treatment processing). Its isoelectric point can be made different during its processing time to yield either a positively charged basic gelatin or a negatively charged acidic gelatin [122]. In some cases, the mechanical strength of gelatin has been increased by cross-linking of the fabricated scaffolds by immersing them into 25% glutaraldehyde for three days at room temperature, followed by keeping in fume hood for 3 h to remove glutaraldehyde [123]. The cross-linked scaffolds exhibited the Young’s modulus value of 33.8 MPa in the axial direction, while the modulus value of natural collagen recorded was 5–10 MPa. Salifu, A.A., et al. have studied the fate of human fetal osteoblast cells on gelatin-hydroxyapatite cross-linked electrospun oriented fiber scaffolds at different hydroxyapatite concentration [51]. Song Ju-Ha, et al. have developed electrospun gelatin nanofiber by water based co-solvent approach [124]. Gelatin can also be incorporated with PCL for the fabrication of nano-fibers for the purpose of bone tissue engineering application [125]. Table 4 shows some mechanical properties of some natural polymers.

### 3.5. Electrospun Scaffolds from Synthetic Polymer

The commonly used synthetic polymers in bone tissue engineering are, polycaprolactone (PCL), poly-lactic acid (PLA), poly(glycolic acid) (PGA), poly(lactic-*co*-glycolic acid) (PLGA), polyurethane (PU), polypropylene, etc. The degradation rate of PLGA can be controlled by controlling the fraction of each PLA/PGA molecular weight. Herein, we discuss some synthetic polymers in brief.

#### 3.5.1. Polycaprolactone (PCL)

PCL is semi-crystalline polyester with glass transition temperature of nearly −60 °C and melting point of 61 °C. Because of multiple advantages like biocompatibility, non-toxicity, biodegradability, and adequate mechanical properties, PCL is widely used in biomedical applications [130]. Furthermore, relatively slow degradation rate and high modulus value makes the PCL mat, a good choice for bone tissue and drug delivery application. The in vivo degradation time for PCL is nearly two years or above [131]. The degradation products of PCL are easily assimilated through metabolic pathways without causing any detrimental effect [132]. In contrary, the degraded products of polylactides and glycolides are acidic and they can affect on stability and performance of protein or other bioactive molecules in the delivery stage.

#### 3.5.2. Polylactic Acid (PLA)

It is a thermoplastic polymer fabricated by the polymerization of lactic acid [133]. It exists in two isomers: poly(l-lactic acid) and poly(d-lactic acid). Polyesters are most commonly used polymer in bone tissue engineering. There are many polyesters that are food and drug administration (FDA) approved. Usually, polyesters can be easily copolymerized with different proportions to tune the degradation rate. PGA and PLA have been shown to promote healing and osseointegration. These are slow delivery carrier for growth factors. Having a low modulus value, PLA must be either copolymerized with polymer having higher modulus or made into a composite with suitable material. PGA and PLA have been copolymerized to control over the degradation rate for a specific application in bone tissue engineering [134].

#### 3.5.3. Polyglycolic Acid (PGA)

Polyglycolic acid (PGA) is a simple linear aliphatic thermoplastic polyester with high crystallinity (46–50%). Its melting point is 225 °C and glass transition temperature of 36 °C [106]. PGA has high modulus (7 GPa) and degrades almost completely within 4–6 months under in-vivo condition. It is good material used for bone tissue engineering application.

#### 3.5.4. Polyethylene Glycol (PEG)

Polyethylene glycol (PEG, Molecular weight less than 20,000 g/mol), polyethylene oxide (PEO, Molecular weight above 20,000 g/mol), and polyoxyethylene (POE, Molecular weight less than 20,000 g/mol) are chemically similar, but only differ by their molecular weight. These are oligomer or polymer of ethylene oxide (-CH_2_-CH_2_-O). Due to the chain length effect, their physical properties are different but exhibit nearly identical chemical properties [135]. Polyethylene glycol is a hydrophilic polyether with a wide range of percentage crystallinity, molecular weight, glass transition temperature, melting point, and degradation rate based on the synthesis technique. Co-polymerization of PEG with other hydrophobic polymers, such as PLA [136], PCL, and PGA have been practiced to enhance the degradation rate and neutralize the acidic products that were obtained by physiological degradation. PEGylation is a process of covalently coupling a PEG structure to another large molecule [137].

### 3.6. Electrospun Scaffolds from Polymer Blends

Natural polymers exhibit good biocompatibility but they tend to display poor processability and mechanical properties as compared to the synthetic polymers. While synthetic polymers have great flexibility in synthesis and modification with good mechanical strength but lack cell affinity due to low hydrophilicity and lack of cell recognition sites. Due to these reasons, natural or synthetic polymer alone cannot meet all of the requirements for tissue engineering. The strategy of blending natural and synthetic polymer such as chitosan/poly-ε-caprolactone can endow good mechanical requirements as well as mimetic ECM topographic cues in tissue engineering [138]. Too, blending of two natural polymers, such as silk fibroin/ collagen, silk fibroin/chitosan [139], or two synthetic polymers, such as PCL/PLA [69], have also been attempted. The commonly blended polymers are PCL/PU, PCL/PLA, silk fibroin/collagen, PCL/gelatin, polydioxanone/elastin, PCL/PEG, etc. [140].

### 3.7. Copolymers in Electrospinning

To get the desired properties that are related to degradation rate, hydrophobicity, percentage crystallinity, and subsequent biological functionality of substrate, researchers make unlike molecules in random sequence to yield polymers, which is called copolymerization. Poly (lactic acid-*co*-glycolic acid) (PLGA) can be used alone or with some other chemicals compound, such as TiO_2_ to composite in the fabrication of scaffolds for bone tissues [141]. The most commonly used copolymers in electrospinning technique for bone tissue engineering are styrene-butadiene-styrene, a triblock copolymer [142], PGA/PLA *co*-polymer [143], etc. This copolymer has been widely used for the encapsulation and release of wide varieties of drugs and bioactive molecules, including TGF-β, BMPs, IGFs, VEGF, NGF, DNA, vancomycin, gentamycin, cisplatin, and so on. Different processing techniques, such as emulsion, freeze-casting, gas foaming, nano and micro particles encapsulation, double emulsion solvent extraction, electrospinning, compression molding, etc. have been encompassed to encapsulate such biomolecules and drugs [144,145]. 

#### 3.7.1. Poly(lactic-*co*-glycolic acid) (PLGA)

PLGA is a very promising material used in the fabrication of bone tissue scaffold due to its biocompatibility, tunable biodegradation rate, and potential to its surface modification, however, its clinical uses is limited due to its relatively poor mechanical properties (especially Young’s Modulus). This necessitates combining PLGA with other polymers, like collagen, chitosan, or bioceramic, such as hydroxyapatite, to enhance its osteoconductivity and mechanical properties [53,146,147,148,149].

#### 3.7.2. Polylactic Acid-*co*-polyethylene Glycol (PLA-PEG)

Polylactic acid-*co*-polyethylene glycol (PLA-PEG) is biodegradable aliphatic polyester that is used as scaffolds in bone tissue engineering. Though its degradation products can be removed via natural metabolic pathways, the degradation product might reduce the pH in local level, which in turn, induces an inflammatory reaction on bone cell damage or death [150]. To sort out the problem, PLA-PEG can be loaded with some other chemical moieties, such as iron oxide nanoparticles [151], human BMPs to accelerate healing, and osteogenesis in vivo [150].

### 3.8. Polymer-Ceramic Composites

Ceramics lack mechanical strength due to brittleness, while polymers lack compressive modulus as compared to native bone tissue. This type of discrepancy can be eliminated or reduced by incorporating the high modulus macro/nano scale constituent ceramics within the polymer matrix. This method develops biodegradable and bioactive polymer-ceramic composites conducive for the use of bone tissue scaffolds [152]. Polymer composites can be formulated by incorporating micro/nano scale hydroxyapatites (HA) or calcium phosphate particles (more commonly, β-TCP) due to their biomimetic and osteogenic properties [153,154]. Besides ceramics, several inorganic/organic constituents, such as carbon nanotubes, nanoparticles, nanospheres, nanoshells, etc. have been dispersed into polymer to improve tensile strength, modulus, and crack resistance of the desired scaffolds [155].

## 4. Clinical Applications of Scaffolds in Medicine

Some natural polymers, such as cellulose, silk, plant fibers, and hairs have been used by humans for medical appliances such as sutures, since beginning [156]. Based on the monomer units, polymerization reaction and formation of *co*-polymers under adjustable concentrations, synthetic polymers may fulfil the structural and mechanical requirements in biomedical applications with tunable physical and chemical properties. Degradable synthetic biomaterials are preferred intentionally to fulfil the specific function in surgical sutures. Electrospun fibers with reactive isocyanate groups are prone to chemical modification with peptides. Layer-by-layer assembly is a suitable method to develop a polyelectrolyte multilayers for potential application in biomedicine [157]. Blood bags are manufactured from polyvinyl chloride (PVC) containing low molecular weight softener, polypropylenes are used for the fabrication of hernia meshes, polytetrafluoroethylene can be used as large diameter vessel substitutes. Hydrolytically degradable aliphatic *co*-polyesters can be used for supporting implants in wound healing [158]. 

In vitro application gives an instinct about the successful setting of any scaffolds in animal model (in vivo), which in turn, becomes a pre-requisite step for clinical applications to assure their applicability [159]. Clinical applications of scaffolds require the regeneration of specific size defects, structural integrity, and sufficient cell penetration, followed by differentiation and proliferation [160]. Yao, Q., et al. have developed three-dimensional (3D) electrospun PCL/PLA blend nanofibrous scaffolds and investigated the in vivo bone formation ability of the substrate in a clinically relevant critical-size cranial bone defect mouse model [69]. Bone morphogenic proteins are food and drug administration (FDA) approved for clinical uses and is found most eminent to induce osteogenic differentiation. Therefore, its incorporation with diverse autografts and allografts are being more popular [159]. Silk fibroin has been used for the regeneration of ligament tissue in animal trial due to outstanding mechanical property and appropriate degradability. Low crystallinity hydroxyapatite/silk scaffold is a good promoter for osteogenesis, as proved by in vitro tests as well as animal trials [161]. The US FDA has approved the silk sutures for its use in soft tissue repair and silk-based biomaterials are being used for the regeneration of musculoskeletal tissue.

Scaffolds serves as a matrix in the reconstruction of tissue and should exhibit some remarkable features, such as ease of handling, sufficient porosity with tunable pore, shape and size for the penetration and diffusion of cells, growth factors, nutrients, along with easy excretion of byproducts from the cells. Furthermore, the scaffold should be biodegradable, bioactive and capable of letting vascularization. However, the mechanical behavior requirements might be different for the different scaffolds. For instance, the mechanical behavior of stents in dentistry and bone tissue are different. Soft and injectable scaffolds are preferred in the pulpodentinal complex and periodontal apparatus by the constrain of small size and difficulty of reaching to the targeted sites [36]. The biomaterials that were used in the fabrication of rigid scaffolds owe great interest in surgery and are aspired to guide the rebuilding of bone and cartilages. Many natural polymers are preferred to construct soft matrices. Collagen showed great potential in bone tissue engineering [162]. Silk fibroin has exhibited cytocompatibility, biogradability, and minimal inflammatory reaction in bone tissue engineering [163]. Synthetic polymers, such as polyhydroxyl acids (PLA, PGA, PLGA), polyhydroxylketones (PCL), polyhydroxybutyrates (PHB), and polyhydroxybutyrate-*co*-hydroxyvalerate (PHVB) nanofibers exhibited better cell growth as compared to their flat films counterparts and are usable for the constitution of rigid scaffolds [164]. Though synthetic or natural polymers are being used to support the growth of osteoblasts and their progenitor cells to recruit their phenotypic expression and differentiation, some bone-bioactive inorganic compounds, such as hydroxyapatite, calcium phosphate, bioactive glasses, and glass ceramics might be the fascinating choices of materials for the fabrication of hard tissues. The electrospun fibers could act as a vehicle for drug carrier. Zamani, M., et al. have studied the drug release in phosphate buffer saline (PBS, pH 7.4) of PCL nanofibers incorporated with metronidazole benzoate. They evaluated the drug loaded substrate for periodontal diseases. In the study of drug release kinetics, a sustained drug released was found to prolonged for at least 19 days, suggesting its use for locally controlled drug delivery in periodontal diseases [165].

Graphene-membrane composites have been designed to use in bone defect regeneration sites to allow for the infiltration of soft tissue cells into the growing cells. It has been established that graphene oxide enrichment of collagen membrane enhances the osteoblastic differentiation process and decreases the inflammation, serving as a good substitute for the conventional collagen membranes [166].

## 5. Current Development and Future Perspectives

Biomaterials of different animal models and approaches have been developed mimicking natural bone hierarchical structure focused on controlling substrate geometry, surface modification, mechanical properties, and biochemical properties [167,168,169]. In animal physiology, systems work in co-relation rather than in independent manner. The fundamental interconnections in bone tissue is that blood vessels promote neurogenesis by supplying oxygen, nutrients, and neurogenic growth factors, while nerve fibers enhance vascularization by providing vasculogenic neuropeptides in a reciprocal manner, which in synergistic turn, facilitates the bone growth and remodeling. Explicitly, brain-derived neurotrophic factor (BDNF) promotes human bone mesenchymal cells (hBMSCs) osteogenesis and neurogenesis. The BDNF may promote osteogenesis indirectly via increased neurogenesis [170]. Therefore, the engineering of neovascularized bone scaffolds based on biomimetic approaches is expected design for clinical applications. 

Better understanding on mechanical cues and signals in specific cellular behavior and differentiation have been focused, which allows for the design and manufacturing of scaffolds resulting into predictable and optimal tissue development upon active mechanical stimulation and fluid perfusion. Scaffolds owning shape memory and conductive materials are also being used to increase the control over the cellular differentiation. Though, the success in the development of biomimicking scaffolds could replace damaged bone from a structural point of view, most of the bone scaffolds might not induce sufficient blood vessels and nerves. It causes a gap between the native bone tissues and developed bone tissue scaffolds in view of integrating and regulating multiple tissue types to recapitulate the complex microenvironment of bone tissues [171]. Bone tissue scaffolds with neurovascularised networks can more accurately mimic the native skeletal tissues and would be more appropriate to regenerate the bone tissues. Quing, L., et al. investigated the fate of brain-derived neurotrophic factor on the promotion of neurogenesis and osteogenesis in human bone mesenchymal stem cells in the bone formation during tissue engineering [170]. 

The technical limitations of traditional electrospinning in bone tissue engineering might be insufficient in the formulation of three-dimensional scaffolds with hierarchical pore structures. There have been many attempts in the fabrication of 3D porous nanofibers scaffolds via layer-by-layer assembly using special binder [172], still more things have to be done to reach up to practical goals. The effect of biodegraded products on human physiology is always a serious concern in tissue engineering. The current approaches are mainly focused on specific tissue formation. At the same time, other associated tissues, like vascular and neural networks, cannot be undermined in a comprehensive application from a technical point of view. Some more recent models have also undertaken into vasculature predictions. Carlier, A., et al. studied the influence of oxygen on the fracture healing process in bone regeneration [173]. The increased interest in electrospun nano/micro fiber loaded with metal oxide nanoparticles, graphene, or other nanocomposites in bone tissue engineering have led to concerns about the risk of both to human physiology and environment. The assessment of potential risk and safety evaluation is mandatory to ensure its specific biomedical uses. Overall, the use of electrospun nano/micro fibers with or without nanoparticles/composites loading deserves to be deeply explored to attain the required structural feature and their precise biological performance.

## Figures and Tables

**Table 1:** Hierarchical scales of bone tissue.

Structure	Dimension Range	Structural Unit/Moieties	Dimension	Scale	Ref.
Macro	Whole bone dimension	Trabecules	Length	1 mm	[50]
			Diameter	0.1 mm	
		Compact (cortical bone)			
Micro	~10–500 µm	Mature osteoclasts		50–100 µm	[41]
		Single trabeculae	Diameter	50–300 µm	[50]
		Haversian system (Osteon)	Diameter	200–250 µm	
Submicro	1–10 µm	Lining cells		1–2 µm	[41]
		Single lamellae	Thickness	3–7 µm	
		Haversian canal		3–7 µm	
Nano	Few hundred nm—below 1 µm	Collagen fibril		500 nm	
Subnano	Below few hundred nm	Apatites plates (HA)	Dimension	2 × 25 ×50 nm	[50]
		Type I collagen	Diameter	3–10 nm	
		Carbonate apatite	Thickness	2–3 nm	

**Table 2:** Physical Properties of Individual Bone Components.

Bone Component	Property	Measurement	Ref.
Large tensile cortical specimens	Young modulus	14–20 GPa	[51]
Microbending cortical specimens	Young modulus	5.4 GPa.	[52]
Osteon lamellar bone	Young modulus	22 GPa	[45]
Osteonal segment (sample with majority of lamellar orientation in the longitudinal direction)	Elastic modulus	12 GPa	[45]
Osteonal segment	Strength	120 MPa	
Osteonal segment	Elastic modulus	5.5 GPa	
Cortical bone	Elastic modulus	5.4 GPa	[51]

**Table 3:** Electrospinning parameter’s influence on nanofiber morphology.

SN	Parameters		Effect on Fiber Morphology	References
1	Polymer property	Polymer	Fiber morphology is specific to polymer used	[86,90,91]
		Molecular weight	Increased molecular mass of polymer might reduce the number of beads. Fiber diameter increases with higher molecular mass of polymer.	
2	Solvent property	Solvent	Solvent used in electrospinning affect on solution spinnability	[92,93]
		Boiling point/vapor pressure		
		spinnability		
3	Solution property			
		Concentration	Increase in concentration of solution increases the fiber diameter (power law relation).	[91,93,94,95]
			Low concentration of solution led to beaded fibers, Intermediate concentration led to good fiber and high concentration led to bimodal fibers and even higher concentration led to a distributed deposition.	
		Conductivity	Increase in conductivity of solution decreases the fiber diameter	
		Viscosity/Surface tension	Formation of an unstable jet as a resultant effect of surface tension and viscosity led to the bead formation [95].	
4	Processing parameter	Spinning voltage	Increase in voltage decreases the fiber diameter and it is strongly correlated to bead formation.	[91,94,96,97]
		Tip-collector distance	Distance effects on complete evaporation of fiber. Too short and too large distances may generate beads. Increased tip-collector distance represents weak electric field. Greater distance to be covered by the fiber and longer flight time favor the formation of thinner fiber.	
		flow rate	Decrease in flow rate decreases the fiber diameter.	
			High flow rate might generate beads. Fiber diameter increases with increasing feed rate.	
5	Ambient parameter	Humidity	High humidity might affect solvent evaporation.	[90]
		Temperature	Increase in temperature decreases the fiber diameter	[87]
6	Supplementary addition	Salt	Addition of salt might help in reduction of beads	[85]

**Table 4:** Mechanical properties of materials in bone tissue engineering.

Polymers/Substrate	Ultimate Tensile Strength (MPa)	Modulus (GPa)	Breaking Strain (%)	Ref.
Bone	160	20	3	[126]
Silk with sericin (*from B. mori*)	500	5–12	10–23.4	[113,127]
Silk without sericin (*from B. mori*)	740	15–17	4–16	[128]
Collagen	0.9–7.4	0.0018–0.046	24–68	[113,129]
PLA	28–50	1.2–3.0	2–6	[113,129]
